# Post-Earthquake Strengthening of RC Coupling Beams with GFRP Wrapping: Experimental Investigation

**DOI:** 10.3390/ma16176040

**Published:** 2023-09-02

**Authors:** Namık Eser, Erkan Töre, İhsan Engin Bal

**Affiliations:** 1 Department of Earthquake Engineering, Disaster Management Institute, Istanbul Technical University, Istanbul 34469, Turkey; 2 Department of Civil Engineering, Faculty of Engineering, Balıkesir University, Balıkesir 10600, Turkey; tore@balikesir.edu.tr; 3 Hanze University of Applied Sciences, 9747 Groningen, The Netherlands; i.e.bal@pl.hanze.nl

**Keywords:** coupling beams, GFRP, preserve seismic performance, urgent strengthening, post-earthquake wrapping

## Abstract

This research aims to address a post-earthquake urgent strengthening measure to enhance the residual seismic capacity of earthquake-damaged reinforced concrete wall structures with coupling beams. The study consists of a series of tests on half-scale prototype coupling beams with various detailing options, including confined with reduced confinement, partially confined, and unconfined bundles, under cyclic loading conditions. The methodology employed involved subjecting the specimens to displacement-controlled reversal tests, and carefully monitoring their response using strain gauges and potentiometers. The main results obtained reveal that GFRP wrapping significantly enhances the seismic performance of earthquake-damaged coupling beams, even in cases where specimens experienced strength loss and main reinforcement rupture. The strengthened beams exhibit commendable ductility, maintaining high levels of deformation capacity, and satisfying the requirements of relevant seismic design codes. The significance of the study lies in providing valuable insights into the behavior and performance of damaged coupling beams and assessing the effectiveness of GFRP wrapping as a rapid and practical post-earthquake strengthening technique. The findings can be particularly useful for developing urgent post-earthquake strengthening strategies for high-rise buildings with structural walls. The method may be particularly useful for mitigating potential further damage in aftershocks and eventual collapse. In conclusion, this study represents a significant advancement in understanding the post-earthquake behaviors of coupling beams and provides valuable guidance for practitioners in making informed decisions regarding post-earthquake strengthening projects. The findings contribute to the overall safety and resilience of structures in earthquake-prone regions.

## 1. Introduction

Structural walls are frequently incorporated into the design of earthquake and wind-resistant reinforced concrete structures. Their presence is vital in minimizing inter-story drifts by providing rigidity to the building system under horizontal loads. However, the efficient transfer of loads between these wall elements is crucial for achieving coupled wall action, thereby further enhancing the structure’s lateral stiffness and ductility properties. This is where coupling beams come into play.

Coupling beams serve as critical components that facilitate load transfer between structural walls. Their primary function is to ensure that the coupled wall system functions cohesively, with the ability to effectively distribute forces and absorb seismic energy during earthquakes. Consequently, the load and deformation capacities of coupling beams significantly impact the energy dissipation behavior of the entire coupled wall system.

In summary, coupling beams play a crucial role in connecting structural walls, promoting coupled wall action, and improving the overall lateral stiffness and ductility characteristics of earthquake- and wind-resistant buildings. By prioritizing ductility in their design and detailing, coupling beams can effectively contribute to the energy dissipation behavior of coupled wall systems, ultimately enhancing the safety and resilience of these structures during seismic events.

Achieving ductile behavior in coupling beams with low aspect ratios (span-to-depth ratio) is commonly accomplished through the implementation of diagonal reinforcement bundles, a detailing technique initially proposed by Paulay and Binney [1]. Subsequent tests have consistently validated that coupling beams with diagonal reinforcement exhibit significantly more ductile behavior compared with conventionally reinforced beams using longitudinal and transverse reinforcement details. This finding is particularly pronounced in the case of short beams with aspect ratios below 2, as demonstrated in studies conducted by Galano and Vignoli [2], Gonzalez [3], Wallace [4], and Han et al. [5].

In the research conducted by Eser et al. [6], a series of tests were carried out on coupling beams designed with consideration of various detailing layouts. The characteristics of these specimens were thoroughly described in the dedicated specimens section. The test results demonstrated that certain detailing variations, such as reducing stirrups, using regional stirrups, omitting transverse reinforcement in the middle span, restraining axial elongation, and anchoring the longitudinal reinforcements into the shear walls, had a significant impact on the coupling beams’ performance. The study emphasized the importance of adhering to sufficient confinement conditions in the design of coupling beams to ensure their proper functionality and performance during dynamic events such as earthquakes and winds.

Various beam structural strengthening systems are widely used. Various systems and techniques are offered for reinforcing beams, particularly those with a lower aspect ratio, where the reinforcement system demonstrated enhanced strength, energy dissipation capability, and deformation capacity compared with the control beam [7]. Additionally, the impact of the aspect ratio on the reinforced beam and the effectiveness of the strengthening system has been reported in [8].

In recent years, the utilization of fiber-reinforced polymers (FRP) for the repair or strengthening of structures has gained notable recognition. This technique encompasses the external bonding of FRP sheets or plates onto reinforced concrete (RC) beams and slabs, as well as the confinement of RC columns. Strengthening using FRP is characterized by its straightforward nature and absence of the need for cumbersome equipment. A multitude of investigations have demonstrated the efficacy of utilizing FRP sheets or plates for the repair and strengthening of RC beams, effectively restoring the concrete members’ structural integrity [9,10,11].

Numerous researchers have undertaken the retrofitting of concrete beams and columns with carbon fiber reinforced polymer (CFRP) [12,13,14,15,16,17,18,19] and glass fiber reinforced polymer (GFRP) [20,21,22,23,24,25] composites to investigate the augmentation of strength, ductility, durability, confinement effects, formulation of design guidelines, and experimental evaluations of these structural elements. While the outcomes derived from diverse investigations concerning the enhancement of fundamental parameters such as strength/stiffness, ductility, and durability of structural members retrofitted with externally bonded FRP composites are indeed promising, they still exhibit several limitations. Consequently, further research is imperative to establish the recognition of FRP composites as a potential comprehensive solution for enhancing structural integrity. FRP repair offers a straightforward approach to bolster both the strength and design longevity of a structure. Due to its notable strength-to-weight ratio and corrosion resistance, this repair method proves especially suitable for deteriorated concrete structures.

CFRPs and GFRPs primarily diverge in the choice of constituent fibers. CFRP employs carbon fibers, as indicated by its name, while GFRP utilizes glass fibers. CFRPs exhibit notably higher strength and a reduced mass due to their lower density. Riyazi et al. [12] conducted tests on six specimens and strengthened two of them by strip-wrapping with CFRP after sustaining damage. The results indicated an increase in shear strength with strip wrapping, but there was a decrease in stiffness. In another study, Honarparast et al. [13] investigated the reinforcement of coupling beams with CFRP using two samples. The first sample, serving as a control, was tested without any reinforcement. The second sample was tested with diagonal CFRP placement on both sides. The strengthened sample exhibited improved strength, energy dissipation capacity, hysteretic behavior, and ductility compared with the control sample. Additionally, Li et al. [14] studied four specimens, one of which was tested without any reinforcement as a control. The remaining three specimens underwent U-type CFRP wrapping. Among them, one had only U-type wrapping, while the other received diagonal CFRP placement in addition to the U-type wrapping. An anchor was added to the last specimen after U-type and diagonal wrapping. The strengthening process enhanced the deformability, strength, ductility, and energy dissipation capacity of the specimens, with the sample featuring diagonal CFRP showing the best performance. Furthermore, the addition of anchoring to the strengthening method extended the deterioration time of CFRP. Overall, the studies mentioned above demonstrate the effectiveness of CFRP (Carbon Fiber Reinforced Polymer) strengthening in enhancing the performance of undamaged coupling beams. The findings highlight the potential of using CFRP materials as a viable solution for retrofitting and improving the seismic behavior of these critical undamaged structural elements.

GFRPs, on the other hand, are chosen due to their low cost, which often leads to their widespread adoption. Many studies [20,21,22,23,24,25] have observed that the use of GFRP wrap generated considerable gains in the strength and ductility of concrete by providing a strong confinement effect beyond a perfect adhesive contact between the concrete substrate and the wrap. The widespread popularity of GFRP can be due to well-known advantages such as a high strength-to-weight ratio and outstanding corrosion resistance. Furthermore, GFRP has a high tensile strength, a favorable stiffness-to-weight ratio, corrosion resistance, electromagnetic neutrality, outstanding fatigue qualities, and precise control over thermal expansion. Because of their accessible availability and the fact that they are a common strengthening application in practice, they can be employed as a quick, effective, and cost-effective reaction strategy after a big earthquake. As a result, in this study, GFRP was chosen as the preferred material for post-earthquake retrofit applications.

Building upon the insights gained from the coupling beam studies, this article focuses on examining the seismic behavior of damaged coupling beam strengthening with GFRP wrapping. Additionally, the article delves into the subsequent retrofitting of test specimens, considering the implications of the findings from the coupling beam studies. By investigating the performance of damaged coupling beams and the effectiveness of GFRP wrapping as an urgent strengthening technique, the research aims to contribute valuable knowledge and practical guidelines for strengthening coupling beams in real-life structural applications. Ultimately, the study seeks to enhance the understanding of seismic response mechanisms in diagonally reinforced coupling beams and advance the development of effective and rapid strengthening strategies to enhance the seismic resilience of high-rise buildings constructed with structural walls.

Given the significance of coupling beams as vital structural elements, they are typically designed with the consideration of potential damage. However, the aftermath of major earthquakes necessitates immediate remedial measures. Surprisingly, there is a lack of existing studies that address emergency response measures for such critical scenarios in the literature. The research is primarily motivated by the urgent need to address such critical retrofitting scenarios. By focusing on the retrofitting of coupling beams that have already experienced damage, this study aims to explore effective and timely measures to enhance their structural integrity and resilience. By examining and developing retrofitting techniques for coupling beams facing potential collapse, it is intended to contribute valuable insights and practical solutions for emergency response and the improvement of seismic performance in high-rise buildings with structural walls. The research seeks to address the challenges posed by the aftermath of severe seismic events, thereby enhancing the safety and stability of these structures during future earthquakes or other extreme conditions.

In previous studies, the approach often involved strengthening undamaged specimens before testing. However, the present study adopted a different perspective by focusing on strengthening coupling beams that had already experienced damage. This choice was motivated by the practical scenarios commonly encountered in real-life building structures, where damage and collapse mechanisms may occur, necessitating immediate retrofitting measures.

This study looks into the fast, effective, and cost-effective retrofitting and response methods that can be used within the context of an emergency retrofit plan, particularly after an earthquake.

- Fast: The term “fast” is used to highlight the urgency and immediate applicability of the proposed post-earthquake strengthening method. In the aftermath of seismic events, rapid intervention is crucial to ensure the safety and stability of structures. The presented study focuses on a solution that can be swiftly implemented to enhance the seismic performance of damaged coupling beams, thereby reducing the time required for critical structural enhancements. The procedure used to improve structural safety while saving time by avoiding the application of repair concrete with epoxy injection and repair reinforcements in the coupling beam body of a damaged structure is referred to as “fast” in this context.
- Effective: The term “effective” underscores the proven capability of the proposed Glass Fiber Reinforced Polymer (GFRP) wrapping technique to significantly preserve the seismic behavior of earthquake-damaged coupling beams, considering energy dissipation and ductility ratio. The presented experimental results unequivocally demonstrate that the GFRP wrapping method enhances ductility and deformation capacity.
- Cost-Effective: The term “cost-effective” emphasizes the economic feasibility of the proposed method. In emergency retrofit scenarios, the availability of an experimental proven solution that achieves notable seismic performance improvements without excessive financial burden is of paramount importance. The application of GFRP, a less expensive material than CFRP, without having to pay for fixing concrete with epoxy injection or replacing fractured reinforcements in the coupling beam body of a damaged structure, is referred to as “cost-effective” in this context.

As a result, a methodological study was conducted in order to provide a rapid intervention using the economical and efficient wrapping technique, while not interfering with the complete removal and reconstruction of the connecting beams between the shear walls or the repair of the damaged reinforcement by removing the concrete. This technique seeks to reduce damage or the danger of collapse caused by aftershocks or further earthquakes of comparable magnitude, as well as to facilitate the ability to resist such events with little structural deterioration. The significance of the presented research is that it indicates a considerable improvement in the strength and stiffness characteristics of the damaged coupling beams, promising a large impact on strengthening earthquake-damaged high-rise structures, which are usually the biggest problem for the authorities and search and rescue teams in the catastrophic aftermath of a large earthquake. The term “improvement in strength and stiffness” used in this context actually refers to the enhancement of the decreased strength observed in earthquake-damaged elements. This signifies a recovery to approximately the initial state. Due to the damage that existed during the initial stages of loading under the impact of an aftershock, the beam would have entirely disintegrated if the quick strengthening method had not been used. However, with the intervention, the term “increased strength and stiffness of the beam” is used to describe a beam that has been strengthened following the damaged condition; an increase over its original state is not intended. The purpose of such post-earthquake strengthening would be to ensure the stability of the structure during aftershocks, an issue valid particularly for high-rise structures with structural walls, until strengthening or controlled demolition works take place. By enhancing the ductility, deformation capacity, and overall strength of these critical elements, the unique application of Glass Fiber Reinforced Polymer (GFRP) wrap provides a practical and efficient solution.

### Importance and Novelty of the Research

In comparison to previous research efforts on reinforcing deficient beams (undamaged, under-capacity) utilizing a range of strengthening systems and methods, the value and novelty of the current work are underlined. While traditional strengthening techniques like as fiber-reinforced polymers (FRPs) are routinely employed, this study uses Glass Fiber Reinforced Polymer (GFRP) for quick post-earthquake (damaged) strengthening of coupling beams. Fabric-reinforced cementitious matrix (FRCM) and steel-reinforced grout (SRG) systems have also been investigated as alternatives to FRPs [26,27]. Compatibility with the chemical, physical, and mechanical properties of the concrete substrate, ease of installation with conventional plastering or troweling processes, a porous matrix structure that allows air and moisture transport, good performance at high temperatures, and ease of reversibility (dismantling) allowing repair without damaging the original structure, particularly in historical buildings, are all features of these systems. Its popularity is growing as a result of the numerous benefits it provides. However, because the non-permanent strengthening approach was not considered, aspects such as high-temperature resistance and porous matrix structure afforded by FRCM and SRG systems were overlooked for the presented study objectives focused on quick strengthening.

GFRP, on the other hand, was chosen because it is more traditional, less expensive, well-known among reinforcement practitioners, and appropriate for speedy post-earthquake retrofitting. Given the critical need of increasing the seismic performance of coupling beams following earthquake events, rapid execution of reinforcement methods was critical. Furthermore, a comparison of GFRP with Carbon Fiber Reinforced Polymer (CFRP) indicates the distinct characteristics that impacted the selection of GFRP for this investigation. While both materials have strong tensile strength and are extensively utilized in reinforcing applications, CFRP has higher mechanical qualities and is therefore better suited for long-term structural retrofitting applications. However, GFRP is chosen over CFRP for this study since we needed a more cost-effective material with a quick response.

## 2. Experimental Investigation

The tested coupling beams in this study were scaled down to a 1:2 scale, making them suitable for use in mid-height buildings and high-rise structures. The aspect ratio of these prototype coupling beams was chosen as 2, representing the ratio of span to section depth. The cross-sectional dimensions of the coupling beams measured 600 mm × 900 mm [23.62 in × 35.43 in]. For diagonal reinforcement, 2 bundles were planned, each consisting of 8-Φ32 bars.

The shear strength of the prototype coupling beams was determined based on the provisions specified in the TSDC-2018 (Turkish Seismic Design Code) [28]. The material properties used for the calculations were as follows: the yield strength of longitudinal reinforcement (fyd) was 420 MPa (60,915 psi), and the compressive strength of concrete (fc’) was 40 MPa (5800 psi). These parameters were critical in determining the structural capacity and performance of the coupling beams during seismic loading.

### 2.1. Specimens

The tests were conducted using prototype beams produced at a 1:2 scale due to limitations in the capacity of the test equipment. In order to ensure similarity between the prototype beams and the full-scale beams, similarity theory requirements were meticulously met. To achieve this, various parameters such as the volumetric stirrup ratio, reinforcement spacing, and diameter in each prototype tie beam were carefully adjusted to be similar to those in the full-scale beams.

Consequently, the test specimens were constructed with dimensions of 300 mm × 450 mm (11.81 in × 17.72 in). The diagonal reinforcements used in the specimens were selected as 4-Φ22 (4-0.866 in), aligning with the scaling requirements to maintain similarity between the prototype and full-scale coupling beams.

In this study, a total of 3 prototype coupling beams were designed, each featuring diagonal reinforcement bundles. Among them, one beam was designed in accordance with the provisions outlined in the TSDC, ACI 318 [29], Eurocode [30]. Notably, the TSDC requires diagonal bundles to extend into the curtains up to 1.5 times the development length, which was carefully adhered to in the test samples. The longitudinal reinforcement was placed with the required anchorage length, aligning precisely with TSDC specifications.

For the other two samples, a more flexible approach was taken in the reinforcement detailing, differing from the strict adherence to regulations. In line with the study’s purpose, a novel and safer reinforcement detailing process was explored by stretching the stirrup wrapping. This design flexibility was incorporated to investigate the effectiveness of alternative reinforcement configurations in enhancing the seismic performance of the coupling beams.

The specimens discussed in the manuscript, which were designed to investigate different levels of confinement and detailing in coupling beams, were as follows:

All specimens were labeled with the prefix “CB2” to indicate their aspect ratio of 2, where “CB” stands for “Coupling Beam”.

1. CB2-UCB: This specimen represented a coupling beam designed without confining stirrups around the diagonal bundles. “UCB” stands for “Unconfined Bundle”, adhering to regulations for coupling beams with unconfined diagonal bundles.
2. CB2-PCB: The diagonal bundles in this specimen were constructed with partial confining, specifically applied to the coupling beam tightening regions situated at a distance of d/2 from the end of the coupling beam (where d is the effective depth of the beam). “PCB” stands for “Partially Confined Bundle”, reflecting the unique confining application.
3. CB2-RCR: In this specimen, diagonal bundles were constructed without any confinement, deviating from the typical recommendations of increased stirrup wrapping for unconfined diagonal bundles as per ACI 318, Eurocode, and TSDC. The confinement was intentionally reduced to explore the lower bounds of these specimens. “RCR” signifies “Reduced Section Confinement Ratio”.

In addition, since CB2 is valid in all samples, it was not used at the beginning of the sample naming in the continuation of the article. There were a total of 3 original test samples. Additionally, damaged samples were quickly strengthened after each experiment was completed, and the meaning “retrofitted” was appended to the end of the original sample name by using the letter R.

In CB2-RCR, the reduction in the confinement ratio implied that the diagonal beam was fully confined, but with a decreased stirrup ratio, while the diagonal bundles remained unconfined. This unique configuration allowed pushing the coupling beams to their lower boundaries, inflicting damage, and then assessing the effectiveness of the potential retrofitting strategies.

**Figure 1:**
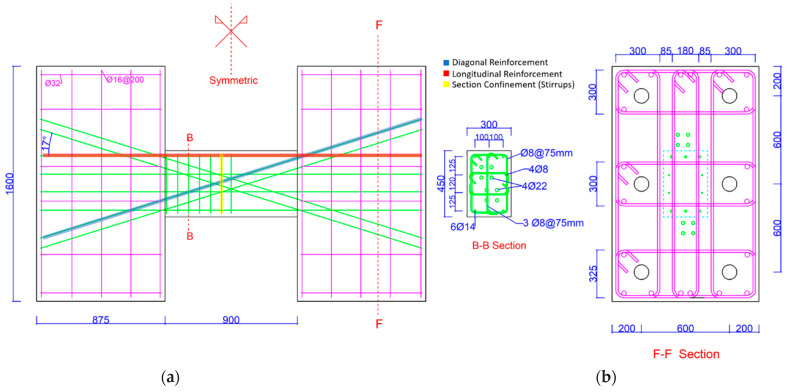
Detailing of CB2-UCB sample and geometry. (**a**) Geometry and details of samples; (**b**) Section details.

**Figure 2:**
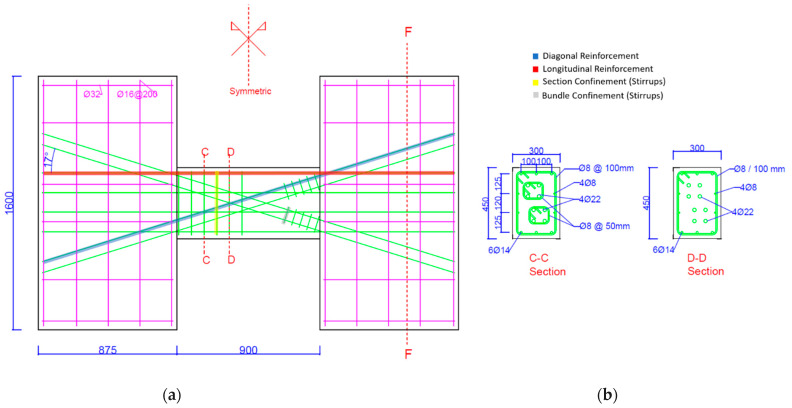
Detailing of CB2-PCB sample and geometry. (**a**) Geometry and details of samples; (**b**) Section details.

**Figure 3:**
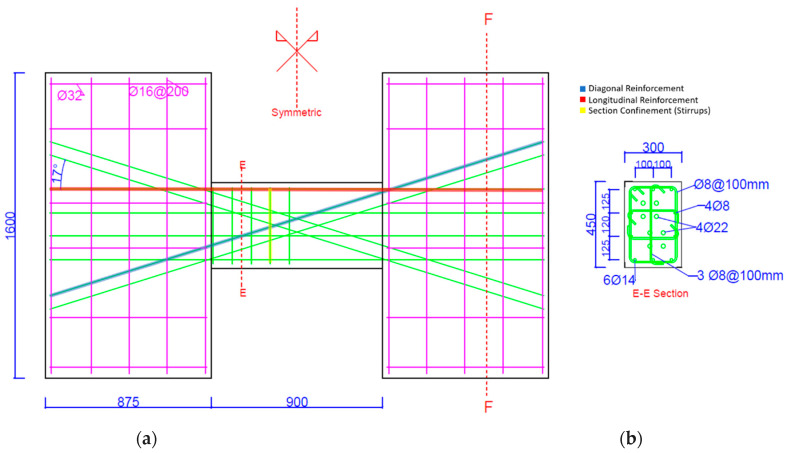
Detailing of CB2-RCR sample and geometry. (**a**) Geometry and details of samples; (**b**) Section details.

The geometry and reinforcement detailing of the tested specimens are summarized in Figure 1, Figure 2 and Figure 3 below.

### 2.2. Materials

Material samples were carefully obtained to determine the mechanical properties of both the reinforcement and concrete used in the study. Concrete samples were subjected to examinations at 7-day, 28-day, and test-day intervals. Likewise, the yield and maximum tensile strengths of the reinforcements were thoroughly tested.

For the CB2-UCB specimen, the concrete strengths at 7-day, 28-day, and test-day evaluations were measured as 31.9 MPa, 40 MPa, and 50.5 MPa (46.2 ksi, 58.0 ksi, and 73.2 ksi), respectively. Similarly, the concrete strengths of the CB2-PCB sample were determined as 35.7 MPa, 40.2 MPa, and 51.9 MPa (51.8 ksi, 58.4 ksi, and 75.3 ksi), respectively, at the corresponding time intervals.

Regarding the last example, CB2-RCR, the concrete strengths at 7-day, 28-day, and test-day assessments were recorded as 41.4 MPa, 48.1 MPa, and 51.4 MPa (60.1 ksi, 69.7 ksi, and 74.5 ksi), respectively.

Furthermore, for the CB2-UCB example, the yield strength of the Φ22 diameter reinforcement was measured as 466 MPa (67.6 ksi), and the maximum strength was observed to be 570 MPa (82.7 ksi). In the case of the Φ14 diameter reinforcement used in the CB2-PCB sample, the yield strength was found to be 464 MPa (67.3 ksi), while the maximum strength reached 579 MPa (83.98 ksi). Finally, for the Φ8 diameter reinforcement employed in the CB2-RCR sample, the yield strength was determined to be 461 MPa (66.86 ksi), and the maximum strength was measured at 592 MPa (85.86 ksi).

Tyfo SEH-25A GFRP [31] material was used to strengthen the damaged samples. Typical test results of the manufacturer for material properties are listed in Table 1. Before the reinforcement process, Teknorep 300 ex repair mortar [32] and Mapewrap 31 T/A epoxy [33] were used for the damaged samples during preparation. Manufacturer typical test results for material properties of these products are shared in Table 2 and Table 3a–c.

### 2.3. Test Setup

**Figure 4:**
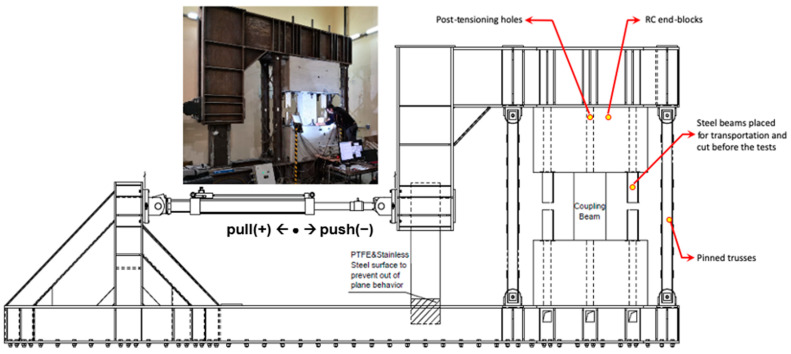
Test setup.

All samples were tested by the setup shown in Figure 4. As the test setup is a self-contained closed system, it does not need a rigid reaction wall. Post-tension is applied so that the upper and lower blocks have a rigid connection. Differential displacements between the steel frame and blocks were monitored throughout the test. Four vertical pin-connected steel columns are attached to both sides of the coupling beam. This is because the coupling beam prevents any rotation at the top block during testing. A rigid box fitted under the L-shaped loading frame is designed to prevent out-of-plane rotation and torsion behavior of the frame and to slide between the lower H profiles. Polytetrafluoroethylene (PTFE), a stainless steel surface, and grease lubrication were used to minimize friction and provide adequate pressure resistance. The lateral load was applied via three horizontal actuators. The lateral load, passing through the mid-span (mid-height) of the test specimen, provided zero moments at the mid-span of the beam.

### 2.4. Loading Protocol

The testing procedure for all samples followed a displacement-controlled reversal test method. The displacement control was achieved by steadily increasing the beam chord rotation. The beam chord rotations were calculated as the ratio of the relative displacement perpendicular to the beam axis at the ends to the clear beam span.

**Figure 5:**
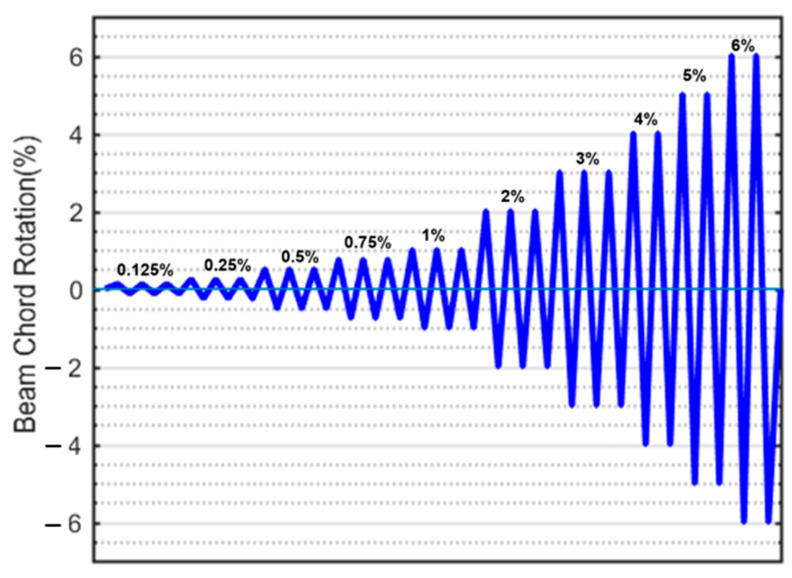
Loading protocol.

The testing procedure involved a combination of displacement-controlled cycles. Under displacement control, three cycles were carried out at every incremental increase in chord rotation, up to a limit of 3%. This limit corresponds to an approximate value for the allowable collapse prevention (CP) limit state specified by ASCE 41-06 [34]. For chord rotations exceeding 3%, two cycles were implemented for each subsequent incremental increase. The loading protocol is visually represented in Figure 5.

### 2.5. Instrumentation

Strain gauges played a crucial role in the experimental setup, providing valuable data on the behavior of the coupling beams during testing. They were strategically placed on longitudinal, diagonal, and transverse reinforcements to monitor their respective stress and deformation responses. Additionally, some strain gauges were affixed to the stirrups of the diagonal bundles and the section stirrups in the middle position.

To gain insights into the stress penetration within the wall, carefully positioned strain gauges along the development length of the diagonal and longitudinal bars were placed. TML-YFLA-5-3L-type strain gauges were utilized in this study due to their reliability and precision.

Data were taken from the strain gauges placed on the original samples. However, strain gauges were damaged as the damage progressed from the coupling beams during the test. Since accurate data could not be obtained from damaged strain gauges, they were not presented comparatively in this study. However, the data for the original samples can be found in [35].

Moreover, sixteen strain gauges were placed at the midpoints of the pinned columns to observe the deformation and resulting stresses arising from the elongation of the connecting beam. These measurements were crucial in understanding the interaction between the coupling beam and the surrounding test setup.

**Figure 6:**
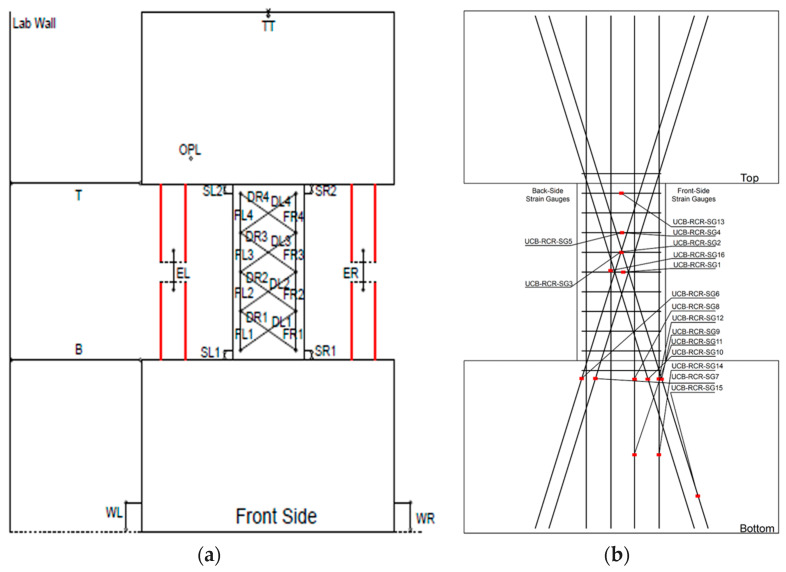
Potentiometer layout (**a**), layout of strain gauges placed on the CB2RCR sample (**b**).

In addition to the strain gauges, potentiometers were employed to measure bending, shear, shear-elongation, shear, and axial deformations along the beam (as depicted in Figure 6). However, it should be noted that data could not be obtained from the specimens after the strengthening process for the 16 strain gauges placed following damage, which rendered them unusable for further testing. Nevertheless, potentiometers positioned on pendulums and on the sample were successfully employed to capture important data during the experiments.

For researchers who want to access and analyze the experimental data, a quick summary of the explanation of the symbols in Figure 6, which describes the configuration of the potentiometer and strain gauges used in the experiment, is provided below [35].

OPL: This symbol was used for out-of-plane measurement.

TT: This was used to observe if there was movement at the top between the experimental setup and the sample.

EL & ER: These were used to measure extension in the left and right, respectively.

DR & DL: These were used to calculate the shear deformation of the coupling beam diagonal left and right, respectively.

FL & FR: These were used to calculate the flexure deformation of the coupling beam left and right, respectively.

SR & SL: These were used to calculate the beam-wall interface slip-extension right and left, respectively.

WL & WR: Potentiometers placed on the left and right of the wall, respectively, at the bottom were used to measure whether there were relative displacements.

T & B: These were used to calculate the relative displacements of the upper and lower points of the beam, respectively.

SG: Strain gauge.

## 3. Strengthening

Prior to the application of the strengthening methods, the specimens damaged as a result of the experimental investigation underwent a fast repair and preparation process. It is critical to note that no repairs were performed to the damaged rebars at this time. Concrete stripping must be done using a concrete breaker in order to regulate all reinforcements and discover their damage, and this is not preferable when working in a damaged structure that requires quick intervention after an earthquake. This deliberate exclusion was motivated by the primary goal of quickly developing an effective post-earthquake reaction strategy for seismically damaged connecting beams.

The purpose for this technique was to investigate a speedy and effective response strategy for earthquake-damaged coupling beams. The next stages of the experimental method were meticulously planned to accomplish this purpose. The properties of the commercial materials used in strengthening are presented in Table 1, Table 2 and Table 3. A commercial repair mortar was employed in the first step of sample restoration. This option was selected in order to effectively restore the structural integrity of damaged sections. Following the careful application of the cementitious repair mortar, the surface was carefully prepared for the subsequent strengthening treatment. The mortar surface was allowed to cure once the initial repair application was completed. To produce a correctly roughened texture, a rigorous cleaning process was undertaken using sandpaper. This phase was crucial to verify that no weak material remained on the repaired surface and that the bonding and adhesion with the FRP composite was optimal.

A commercial epoxy repair mortar was then utilized to remove fracture gaps produced in other damaged regions of the samples to improve adhesion and contact qualities. After carefully cleaning and repairing the substrate, the emphasis moved to the installation of the strengthening system. A commercial epoxy was applied to areas targeted for strengthening to provide a strong adhesive substrate for subsequent strengthening layers. Two layers of GFRP were used during the strengthening process. This GFRP material is thicker than its carbon counterpart, allowing application to rough and uneven concrete surfaces with higher tolerance.

**Figure 7:**
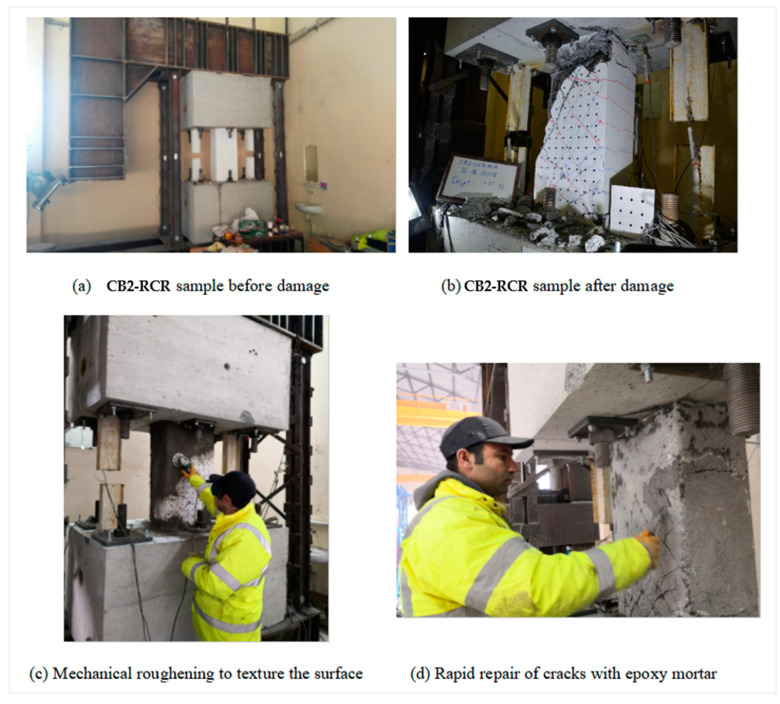
Stages of inflicting earthquake damage and strengthening on the CB-RCR specimen.

**Figure 8:**
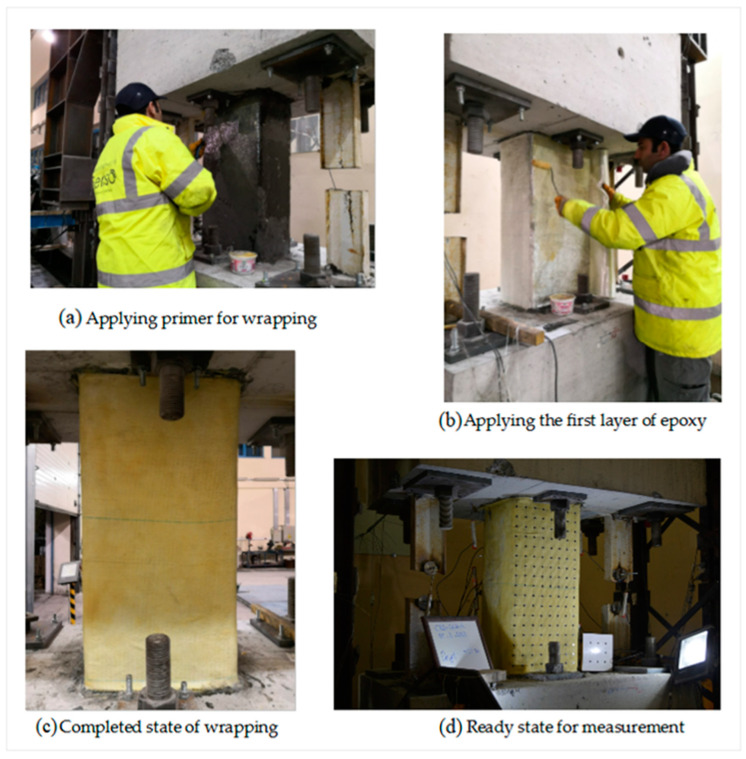
Stages of wrapping the CB2-RCR specimen with GFRP and preparing it for testing.

Figure 7 and Figure 8 show the thorough execution of these preparatory stages, which seek to achieve a smooth integration between the restored substrate and subsequent strengthening materials. These graphic representations provide a full view of the step-by-step process of specimen repair and strengthening underpinning the applied experimental technique. Below, details of each stage of the strengthening application are given:

Step 1: Surface Preparation

The process is initiated by thoroughly cleaning the concrete beam’s surface to eliminate any dust, debris, or contaminants.

To enhance the bonding between the concrete and GFRP, methods such as sandblasting or mechanical roughening were applied to texture the surface.

FRP material cannot be applied on rough edges; thus, a rounding at the beam edges, with a radius of 30 mm, was applied.

Step 2: Application of Bonding Agent

A bonding agent or primer was carefully applied to the prepared concrete surface. This step significantly improved the adhesion between the concrete and GFRP.

Step 3: Cutting and Arranging of GFRP Fabric

GFRP fabric was cut into specific shapes and lengths in accordance with the design requirements. The GFRP fabric was then positioned and aligned on the concrete beam.

Step 4: Mixing and Applying of Resin

Following the manufacturer’s guidelines, two-component epoxy resin was prepared. The epoxy resin was spread onto the designated area of the concrete surface where the GFRP would be placed. GFRP fabric was then positioned onto the wet resin, using brushes and rollers to ensure proper impregnation and the removal of air bubbles. Additional resin was applied to achieve complete saturation of the GFRP.

Step 5: Wrapping and Compaction

GFRP was tightly wrapped around the concrete beam, adhering to the prescribed wrapping pattern and overlap. To ensure optimum contact between the fabric and concrete, rollers and other tools for compacting the GFRP were used.

Step 6: Curing

The epoxy resin was allowed to cure under specified conditions, including temperature and humidity, as per the recommended curing time.

Step 7: Finishing Touches

Upon full curing of the GFRP and resin, a thorough inspection was conducted to verify proper adhesion and coverage. Any excess GFRP fabric was trimmed if necessary.

It should be noted that in real-life applications, a protective coating or finish may be applied to the GFRP surface, enhancing its durability and resistance to environmental factors such as fire.

**Figure 9:**
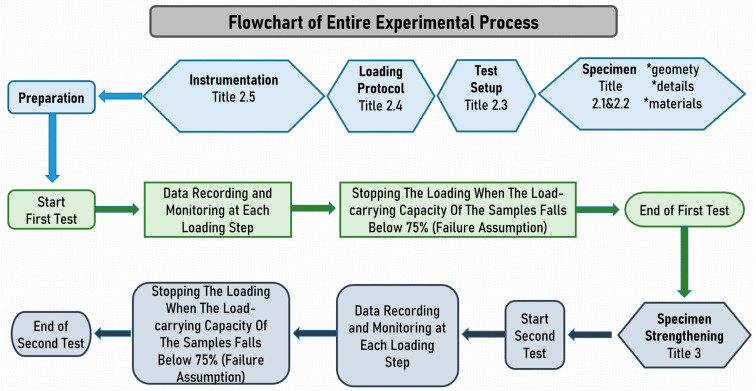
Flowchart illustrating sequential phases of experimental studies.

The schematic representation of the step-by-step progression of the conducted experimental studies is visually presented in Figure 9, mirroring the sequential phases as outlined. Commencing from the inception of initial specimens and extending through the preliminary seismic assessments, subsequent phases encompassing the reinforcement of damaged samples and their subsequent re-subjection to seismic testing are comprehensively depicted. This visual depiction serves as an illustrative guide, offering a concise overview of the entire experimental process, from the fabrication of the initial specimens to the ultimate phase of post-reinforcement seismic evaluations.

## 4. Experimental Results and Discussion

**Figure 10:**
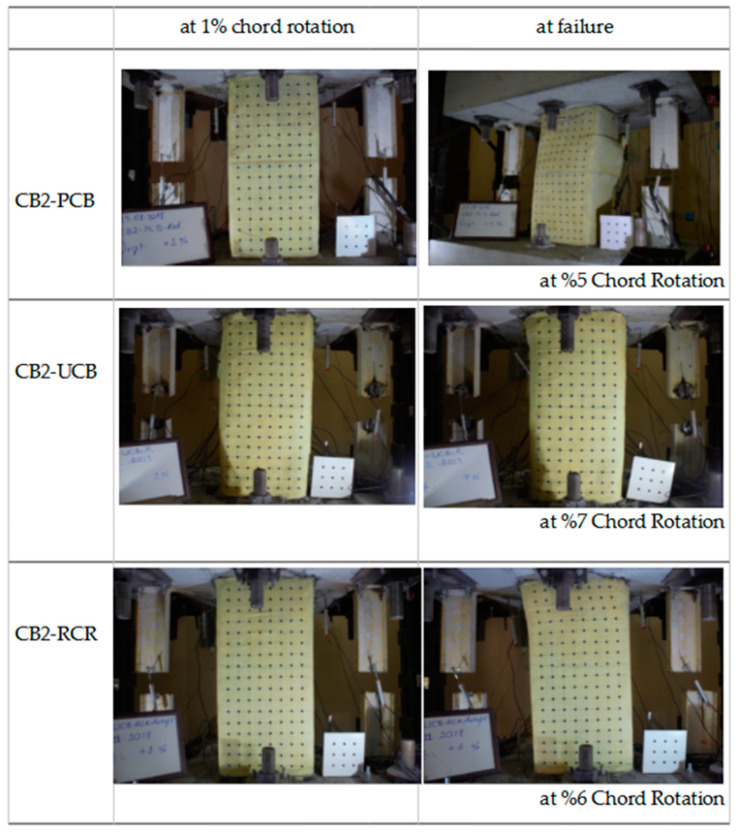
Conditions of the samples during the tests.

In this section, the test results will be presented and discussed. The variations in shear vs. chord rotation, axial elongation, and effective stiffness will be discussed to gain insights into the findings. Although a substantial amount of data were collected, only the most significant results will be highlighted here, as previously mentioned in the preceding section. All experimental studies have been uploaded to an online platform and will be made freely available upon request [35]. It is important to note that chord rotation was utilized for the deformation measurements in the samples examined in this study. Chord rotation is a widely accepted metric for assessing deformations in coupling beams. Additionally, a decrease in strength of approximately 25% from the maximum attainable strength is considered to define the failure mechanism of the coupling beam. Figure 10 displays the 1% chord rotation and collapse mechanism of the tested samples.

Before moving on to the evaluation of the test results, it would be appropriate to start a discourse on the concept of axial elongation. Axial deformation relates to the longitudinal elongation that occurs in coupling beams, which distinguishes them from conventional beams. The amount of elongation per unit length of the beam gives the axial deformation. This deformation, explained through analysis of crack patterns and principles of structural mechanics, is observed by the formation of dense diagonal shear cracks (see Figure 7).

Large, inclined shear cracks can be observed in the coupling beams, according to the crack pattern in Figure 7. They were created between inclined shear cracks and diagonal concrete struts. From the perspective of static balancing, the longitudinal component of the compression pressures pushes the wall panels, lengthening the coupling beams, while the transverse component resists the applied shear load. From a kinematic perspective, the diagonal concrete struts rotate around the compressive corners of the coupling beam as the lateral translation of the beam increases, leading to axial elongation of the beam. But this explains why the amount of axial elongation is larger the lower the span/depth ratio of the beam. The axial elongations were measured through the utilization of potentiometers located on pendulums, as illustrated in Figure 6. These potentiometers were indicated by the symbols EL and ER, representing “Elongation Left” and “Elongation Right,” respectively [36].

Since the axial deformation changes according to the beam’s aspect ratio, the results are presented as axial elongation (mm) instead of axial deformation, with directly measured values, in order to create a numerical level for engineers in practice in these test samples with an aspect ratio of 2.

**Figure 11:**
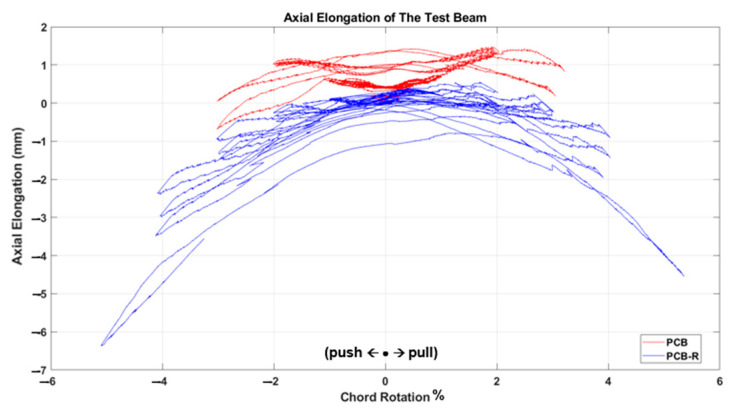
Comparison of the axial elongation of PCB specimen before and after strengthening.

**Figure 12:**
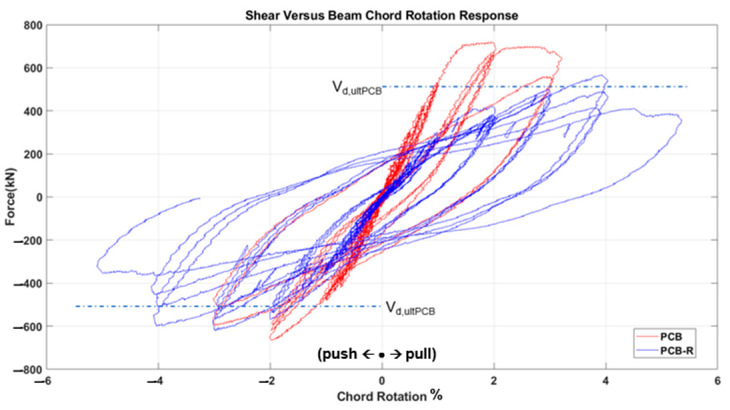
Comparison of pre- and post-strengthening shear force and chord rotation of PCB specimen.

**Figure 13:**
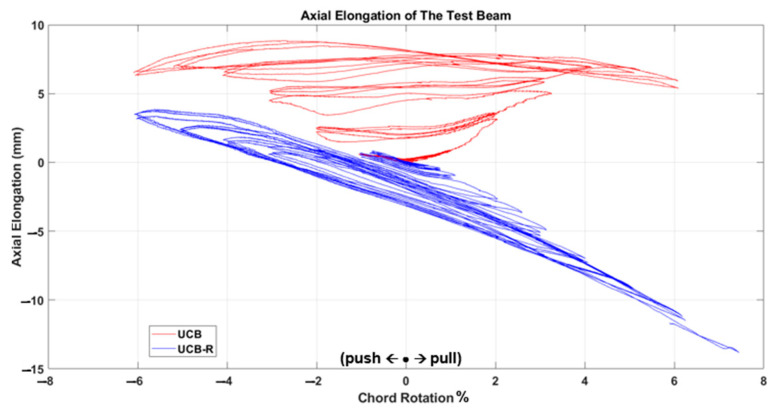
Comparison of the axial elongation of UCB specimen before and after strengthening.

**Figure 14:**
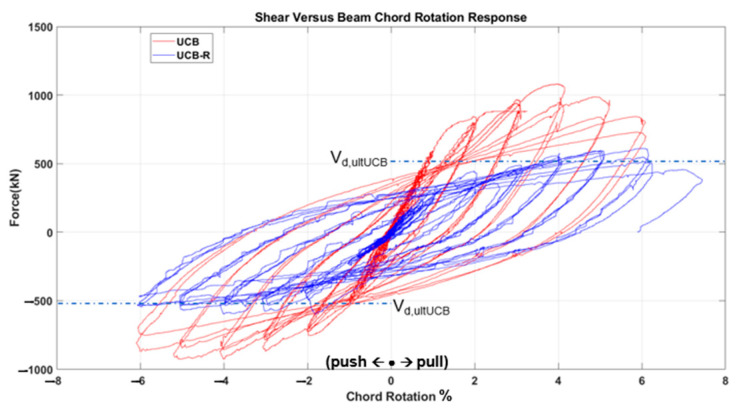
Comparison of pre- and post-strengthening shear force and chord rotation of UCB specimen.

**Figure 15:**
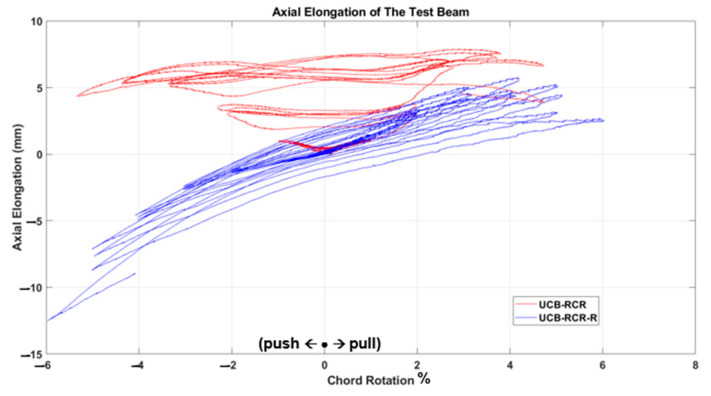
Comparison of the axial elongation of UCB-RCR specimen before and after strengthening.

**Figure 16:**
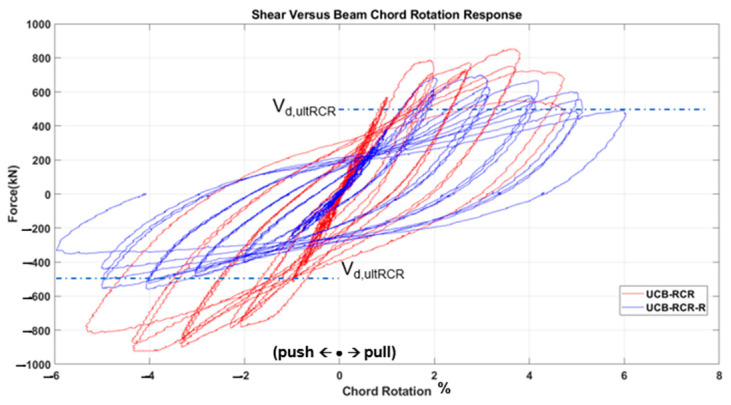
Comparison of pre- and post-strengthening shear force and chord rotation of UCB-RCR specimen.

The test results, depicted in Figure 11, Figure 12, Figure 13, Figure 14, Figure 15 and Figure 16, include the axial elongation and shear force distribution via chord rotation. Notably, the axial elongation values predominantly exhibited negative behavior. This can be attributed to several factors associated with the repair and retrofitting process of the damaged beam:

Closure of Cracks: The use of repair mortar without epoxy injection has not resulted in the closure of cracks in the beam, which had previously experienced high drifts and wide crack formation. During cyclic loading, the main surfaces of the beam had come into contact with each other, leading to the closure of the cracks.

Plastic Elongation and Buckling: The observed plastic elongation in the reinforcement and the presence of buckling could have been influenced by the advanced level of buckling experienced in the previous experiment. The buckling phenomenon might have reached an advanced stage during the subsequent tests.

Damage Patterns: Further examination of the damages from the previous test revealed distinctive behaviors in different beam types. For instance, in the case of the Primary Coupling Beam (PCB), negative elongation was observed throughout the entire cycle. This behavior can be primarily attributed to the PCB’s loss of capacity due to concrete and stirrup rupture in the middle region. Similar damage patterns of negative elongation were observed in Reduced Section Confinement Ratio (RCR) and Unconfined Bundle (UCB) beams, where reinforcement buckling and concrete rupture occurred in the end regions. Negative elongation occurred when the concrete ruptured and reinforcement buckling took place, while limited positive elongation was observed in the other direction. These damage patterns observed in the experiments align with those typically observed in the failure stage.

Behavioral trends observed in the axial elongation graphs of two samples, UCB-R and UCB-RCR-R, which seem different at first glance, can be attributed to the fact that the damage occurred on different sides of the beam. All samples are tested under cyclic loading and the samples are symmetrical within themselves. However, the occurrence of damage on the positive or negative side with each charge cycle is a random event for symmetrical samples. According to the authors, the difference in tendancy in the results does not indicate a behavioral difference.

In conclusion, the negative axial elongation (shortening) values observed in the tests can be attributed to the closure of cracks, plastic elongation, and buckling effects in the beams under cyclic loading. These findings provide valuable insights into the behavior of the repaired and retrofitted coupling beams, shedding light on their structural response and potential failure mechanisms. Understanding these factors is crucial for developing effective retrofitting strategies to enhance the seismic resilience of reinforced concrete structures.

When examining the three fundamental properties of hysteresis response, ultimate strength, initial stiffness, and degradation, the following observations were made for each specimen.

- Partial Confined Bundle (PCB) Specimen:
  ➢ The initial strength of the PCB specimen was higher than the strength after strengthening, but the rate of strength reduction decreased after damage (i.e., a more ductile behavior was obtained).
  ➢ The initial stiffness decreased by approximately 50% after damage at 1% chord rotation.
  ➢ The degradation increased rapidly after reaching the displacement corresponding to ultimate strength as the confinement effect had already diminished in the first test state before strengthening. The strengthened specimen, on the other hand, still showed considerable ductility by exceeding the target displacements.
- Unconfined Bundle (UCB) Specimen:
  ➢ The strength of the UCB specimen was higher initially, before becoming damaged in the previous test campaign. After the strengthening, the strength reached approximately half the strength of the original (i.e., undamaged) specimen at similar chord rotation levels.
  ➢ The initial stiffness again decreased by roughly 50% at the 1% chord rotation value after damage.
  ➢ The degradation tendencies were similar to the undamaged state in the strengthened specimen, but further accelerated with the diminishing wrapping effect at the end of the test.
- UCB-Reduced Confinement Ratio (RCR) Specimen:
  ➢ The strength was stable in the first cycles, but as the number of cycles increased, the strength of the strengthened specimen decreased further due to damage internally occurring behind the wrapping layer. This was observed during the test by the authors.
  ➢ There was roughly a 40% difference between the initial stiffness value at the 1% chord rotation value.
  ➢ The strength degradation was higher in the initial test specimen with increasing chord rotations, while in the strengthened specime, n higher chord rotation levels could be achieved.

In general, the strengthened specimens exhibited lower strength and stiffness as compared to the initial undamaged specimens. It is worth noting that the strength values observed in the strengthened specimens were still around the ultimate design strength values (V_dult,UCB_: 506.6 kN, V_dult,PCB_: 514.7 kN, V_dult,UCB-RCR_: 526.2 kN) and the chord rotation values reached and even passed a minimum of code-enforced 3% drift in all specimens. Furthermore, all three specimens presented a relatively high ductility until collapse. Therefore, it is believed that this urgent strengthening technique holds the potential to effectively enhance the seismic resilience of coupling beams subjected to forces up to the collapse drift, significantly increasing the safety of the overall structure in the post-earthquake situation.

On another note, the constructive longitudinal reinforcement of the connecting beams with diagonal bars acts as an anchorage for the transverse reinforcement, which is critical for shear resistance. However, because of the existence of longitudinal reinforcements, the core concrete within the bundle becomes more prone to crushing and buckling during further chord rotations, particularly at the beam’s ends. As a result, ACI 318-19 and Eurocode 8 specify that the longitudinal reinforcements only serve as anchors to the stirrups and that there is insufficient anchorage length to the shear walls. Lower chord rotation levels were obtained in the original specimens where the stirrup confinement efficiency was poor. The angle of the diagonal reinforcement, the total reinforcement area, the mechanical properties of the reinforcement, and the contribution from the longitudinal reinforcement if anchored into the shear walls all contribute to the shear capacity of the specimens. However, because confinement reinforcement is present, diagonal and longitudinal reinforcements can execute their functions without buckling.

**Figure 17:**
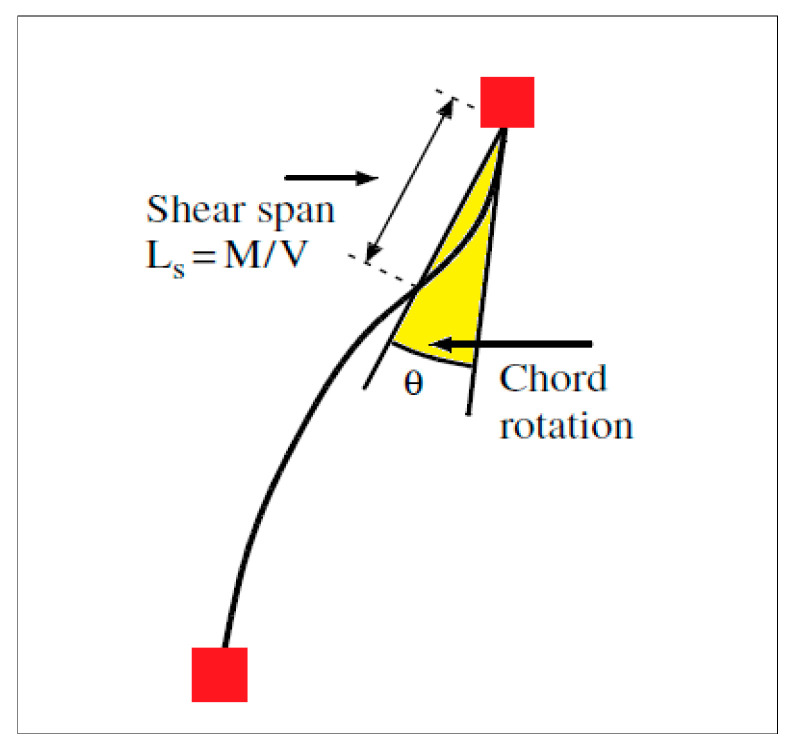
Chord rotation and shear span relation of the beam element.

The graph below depicts the definition of the shear capacity and coupling beam chord rotation (Figure 17). The preservation of shear capacity in increasing chord rotations without a significant decrease in maximum shear capacity is a sign of a successful post-earthquake strengthening, meaning that the wall-to-wall interaction via the coupling beams can still hold for further displacements demands in the aftershocks. For instance, the diagonal rebars, which are responsible for directly resisting the shear forces acting on the beam, but are also susceptible to buckling, are successfully held by the confinement provided by the GFRP wrapping, hence retaining the shear capacity.

### 4.1. Energy Dissipation

**Figure 18:**
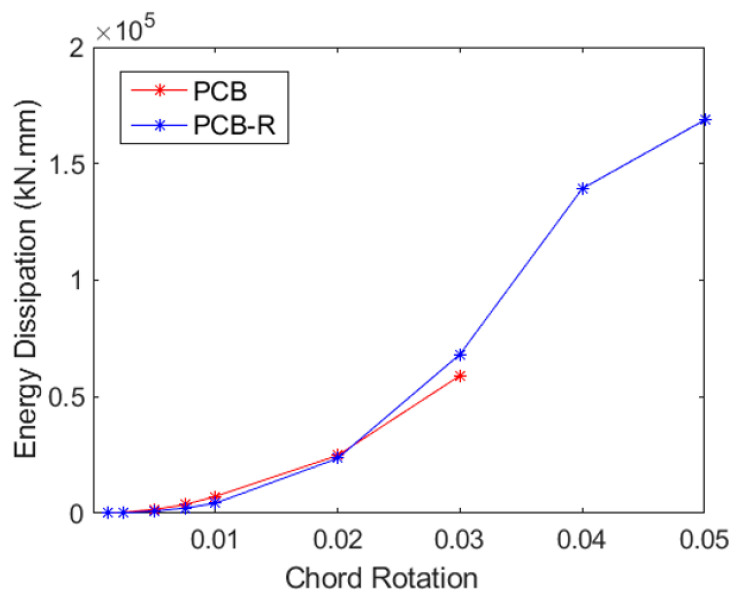
Cumulative energy dissipation for PCB and PCB-R specimens.

**Figure 19:**
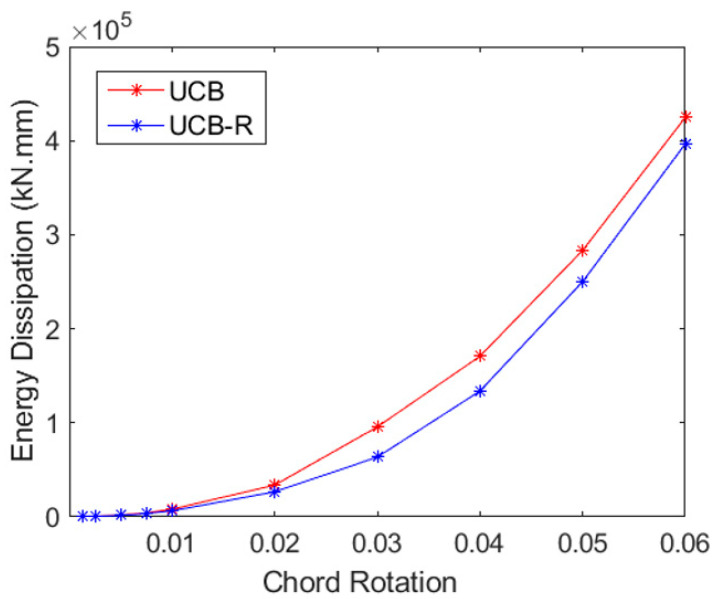
Cumulative energy dissipation for UCB and UCB-R specimens.

**Figure 20:**
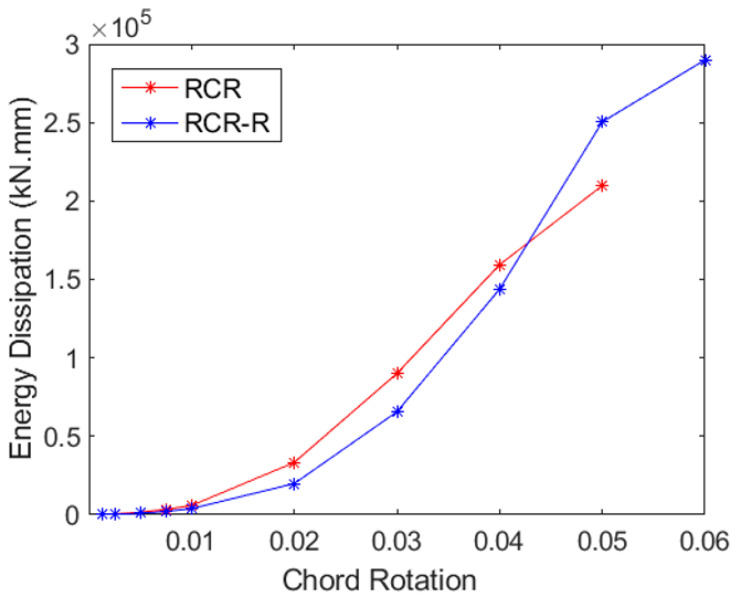
Cumulative energy dissipation for RCR and RCR-R specimens.

Evaluations of displacement and curvature ductility provide important information on the overall load-deformation capacity, while energy-absorbing capacity analysis sheds light on the structure’s potential to withstand seismic pressures. The total energy dissipation in each of the three samples is shown in Figure 18, Figure 19 and Figure 20. The region enclosed by the particular load–displacement curve during a loading cycle provides an approximation of the amount of energy lost during that cycle. The total energy expended across all cycles up to the given displacement is depicted in Figure 18, Figure 19 and Figure 20 as cumulative energy.

When the CB2-PCB (Coupling Beam—Partially Confined Bundle) sample is examined, it is seen that the chord rotation rate, which can reach up to 3% without GFRP wrapping, increases to approximately 5% after earthquake damage, after wrapping with GFRP. The specimen encased in GFRP also takes in around 5% more energy during the 3% chord rotation cycle. In this situation, it appears that the GFRP wrapping considerably increases the lack of confinement effect brought on by the coupling beam’s absence of stirrups.

When the CB2-UCB (Coupling Beam—Unconfined Bundle) sample is examined, it is seen that the chord rotation ratio, which can reach up to 6% without GFRP wrapping, reaches the same level after earthquake damage and after GFRP wrapping. However, the specimen wrapped with GFRP absorbs approximately 15% less energy during the 6% chord rotation cycle. In this context, although the almost same confinement effect was achieved with GFRP due to cracks and reinforcement damage in the web, which occurred despite the tight stirrup wrapping in the beam in the first case, the energy absorption capacity could only approach its undamaged state, not higher.

Evaluating the CB2-RCR (Coupling Beam—Reduced Section Confinement Ratio) sample, it can be observed that the deflection rate, which can reach up to 5% without GFRP wrapping, increases to about 6% following post-earthquake damage GFRP wrapping. Also, the specimen wrapped with GFRP absorbs approximately 25% more energy at 5% chord rotation cycles and beyond. This increase significantly resulted in 5% chord rotation instead of 6% due to the reduced confinement effect in the initial state of the beam compared to the UCB sample. However, the strengthening sample reached up to 6% chord rotation when the confinement effect was largely recovered with GFRP. As a result, it has increased its capacity to absorb energy. The contribution of stirrups on ductility and energy dissipation is evident in diagonally reinforced coupling beams based on these results, and it is rather difficult, if possible at all, to exceed their contribution by simply applying GFRP wrapping.

### 4.2. Ductility

This study focuses on the significance of evaluating seismic retrofitting strategies to enhance structural performance and reduce damage. In this context, a key aspect emerges in the analysis of ductility index values, particularly illuminating the effectiveness of Glass Fiber Reinforced Polymer (GFRP) wrapping. Detailed ductility behaviors of different coupling beam configurations, namely CB2-UCB (Coupling Beam—Unconfined Bundle), CB2-PCB (Coupling Beam—Partially Confined Bundle), and CB2-RCR (Coupling Beam—Reduced Section Confinement Ratio), are thoroughly examined from the perspective of specific pre- and post-GFRP wrapping ductility values.

The ductility index values presented in Table 4 reflect the ductility performance of each configuration. These values encompass the specimens’ performance before and after wrapping application, providing a comprehensive assessment of the impact of GFRP wrapping on ductility enhancement.

Initial observations regarding pre-GFRP wrapping ductility values are intriguing. In the case of CB2-UCB, the pre-GFRP wrapping ductility value of 4.44 increases slightly to 4.47 post-GFRP wrapping. Notably, this reveals that the specimen, which had experienced substantial ductility loss due to earthquake damage, regained its initial ductility level through GFRP wrapping, achieving the intended purpose of the wrapping.

For the CB2-PCB configuration, the pre-GFRP wrapping ductility value records as 2.23. Impressively, post-GFRP wrapping, this value surges significantly to 4. This substantial increase underscores the notable impact of wrapping on enhancing ductility. The initial lower ductility value was attributed to the wider spacing of section stirrups and the partial absence of stirrup diagonal bundles. The substantial augmentation of ductility values post-wrapping indicates a successful mitigation of these deficiencies, nearly doubling the initial ductility level.

Similarly, the CB2-RCR configuration exhibits intriguing behavior. The initial pre-GFRP wrapping ductility value is 3.5, experiencing a very slight elevation to 3.51 post-GFRP wrapping. This slight change, in line with other observations, underscores the intricate relationship between damage, GFRP wrapping, and overall ductility performance. The ductility level that was reduced due to damage is maintained through wrapping. When the results are analyzed, it can be claimed generally that the PCB sample’s ductility ratio increased, whereas the ductility ratio in the other two samples was retained as compared to those of the corresponding initial (i.e., undamaged) specimens.

In conclusion, the efficacy of GFRP wrapping in mitigating damage-induced reductions in ductility, even, in some situations, surpassing original undamaged ductility levels, is evident. These findings underscore the adaptability of GFRP wrapping to address deficiencies arising from design choices and restore structural performance.

In summary, the outcomes of this study underscore the potential of GFRP wrapping in reducing the effects of damage and enhancing ductility levels.

### 4.3. Residual Deformation

Furthermore, investigating residual drift offers essential knowledge regarding the structure’s ability to return to its original position after experiencing seismic loads. Since the process of strengthening was done after the earthquake damage, the beam was strengthened underresidual displacement in the position where the force on the loading pistons was zero. This illustrates the situation where damaged beams displace irreversibly as a result of the earthquake. The examination of crack propagation and failure models contributes significantly to the understanding of potential vulnerabilities and modes of failure. After the beam was strengthened, cracking propagation could not be monitored anymore, but failure occurred with the GFRP composite rupturing as a failure mechanism from the same locations, much like how the initial damage in the original sample developed, suggesting similarities between the original and the strengthened specimens in terms of internal stress developments.

Table 5 displays the residual deformation values for each sample before and after the GFRP wrapping was applied. In order to restore their original geometry even in the presence of residual deformations (under zero load conditions), these specimens were restored with cementitious structural repair mortar. With careful and exact GFRP wrapping techniques, the performance levels of the samples were successfully returned to those seen prior to damage. Additionally, it is clear that the quick strengthening method may be used in the majority of situations to repair an earthquake-damaged structure given the efficiency of the GFRP wrapping method on a UCB sample up to 30 mm (after 6% chord rotation).

### 4.4. Effective Stiffness

**Figure 21:**
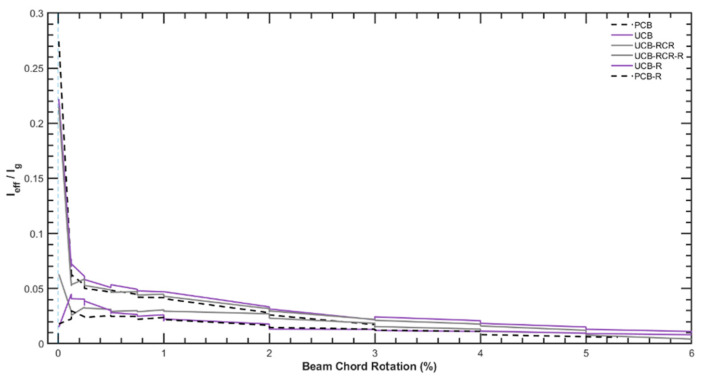
Comparison of I_eff_/I_g_ changes of samples before and after reinforcement.

When analyzing the effective stiffness values, Figure 21 indicates that the values at the end of the tests before strengthening are predominantly maintained during the tests conducted after the strengthening process. This observation suggests that the strengthening measures applied to the specimens have effectively preserved their overall stiffness characteristics, especially after 2% chord rotation. This finding suggests that the retrofitting measures, such as GFRP wrapping and repair mortar application, effectively maintain the overall stiffness of the coupling beams even after undergoing cyclic loading and experiencing damage. The strengthening technique has successfully enhanced the structural stiffness, contributing to the improved performance and seismic resistance of the coupling beams throughout the test cycles. This finding further supports the feasibility and efficacy of the proposed strengthening approach in maintaining the structural integrity and enhancing the safety of the coupled wall systems.

In this context, the effectiveness of the reinforcement system has been assessed through various critical parameters, including energy absorption, displacement, curvature ductility, residual drift, crack propagation, and failure models. These comprehensive evaluations play a vital role in understanding the system’s performance under seismic conditions.

The ability to retain effective stiffness is crucial for the structural performance of coupling beams, as it ensures that the beams can continue to provide the necessary lateral stiffness and ductility during seismic events. By demonstrating the capability to preserve effective stiffness through strengthening, this study further supports the feasibility and reliability of the proposed method in enhancing the seismic resilience of reinforced concrete structures.

The initial stiffness of each coupling beam is around 25% of the gross elastic stiffness (EI), with an effective stiffness (Ieff) of 0.08 EI at the yield rotation (1.0% rotation). The effective secant stiffness values corresponding to the ASCE 41-19 limit states are approximately 0.15 EI at Immediate Occupancy (0.6%), 0.075 EI at Life Safety (1.8%), and 0.05 EI at Collapse Prevention (3% rotation). The effective stiffness ratio (Ieff/I) does not differ considerably across the three layouts. The authors recommend evaluating the effective stiffness value of the samples reinforced with GFRP as 3% to 5% EI when applying a modeling approach in establishing structural safety after an earthquake, given that quick strengthening without epoxy injection is used in damaged situations.

Within the scope of this research, an extensive investigation was conducted to comprehensively evaluate the efficiency of the strengthening system in terms of deformation capacity, a key indicator of seismic performance of RC coupling beams. The analysis revealed that chord rotations reached levels of 4% and above in the strengthened specimens. This noteworthy achievement becomes particularly significant when considered together with the outcomes of undamaged specimens. A comparative analysis demonstrated that the specimens with inadequate stirrup confinement exhibited a notable enhancement in post-yield capacity within the pre-yield displacement range, owing to the utilization of the proposed strengthening method. In contrast, specimens with sufficient stirrup confinement maintained similar levels of capacity as their undamaged counterparts. This observation underscores the pivotal role of proper stirrup confinement in ensuring augmented deformation and ductility capacities, crucial aspects influencing the seismic performance of reinforced concrete elements.

## 5. Conclusions

This study investigated the seismic behaviors of earthquake-damaged reinforced concrete coupling beams strengthened with Glass Fiber Reinforced Polymer (GFRP), aiming to present a rapid and effective post-earthquake strengthening solution. Through experimental tests on half-scale diagonally reinforced concrete coupling beams with various detailing options, including reduced confinement ratio, partially confined, and unconfined bundles, the performance of the rapid GFRP wrapping method was thoroughly evaluated under cyclic loading protocols.

The experimental results unequivocally demonstrate that GFRP wrapping significantly improves the seismic performance of earthquake-damaged coupling beams, even in the presence of more than 25% strength loss, which means failed specimens. The strengthened beams exhibit commendable ductility, maintaining high levels of deformation capacity after strengthening, although there is a notable reduction in overall stiffness. Moreover, the chord rotation level of the strengthened beams complies with the stringent requirements of ACI 318, Eurocode 8, and the Turkish Earthquake Code.

This study underscores the effectiveness of GFRP as an efficient solution for enhancing the post-earthquake performance of damaged coupling beams. The strengthened beams exhibit commendable ductility and high-level deformation capacity. By demonstrating the capability to preserve effective stiffness through strengthening, this study further supports the feasibility and reliability of the proposed method in enhancing the seismic resilience of RC structures. The significance of quantifying seismic performance improvements in high-rise building structures using GFRP reinforcement should also be considered. Structural safety can be controlled during permanent reinforcement work in the structure by strengthening the application and control of the presented method.

The significance of quantifying seismic performance improvements in high-rise building structures using GFRP reinforcement should also be considered. To assess the level of seismic enhancement from GFRP-strengthened coupling beams and the effect of GFRP reinforcement on seismic behavior, two easily controllable indicators are recommended. Natural Frequency Shift (tracking natural frequency shifts) and Experimental Modal Analysis (determining changes in Mode shapes and frequencies) are two potential indicators. The noticeable rise in the initial stiffness of the reinforced beam seen in Figure 21 can be utilized as a concrete indicator, as can the frequency shift and mode shape change. Structural safety can be controlled during permanent reinforcement work in the structure by strengthening the application and control of the method with these proposed indications.

After conducting the tests on the RC specimens, their failure mechanisms were carefully examined. Upon evaluating the GFRP-strengthened specimens by opening the concrete cover at the end of the test, the authors observed rupture in the longitudinal and transverse reinforcements in regions experiencing high stress or deformation demands. This observation provided valuable insights into the behavior of the strengthened specimens under load and offered a clearer understanding of the performance of GFRP materials in enhancing the structural integrity of coupling beams. The influence of section confinement level on the performance of the coupling beams has been established as a key factors that dictates the success of the strengthening strategy.

High-rise structures are far more expensive in demolishing and take longer to strengthen as compared to low-rise buildings. They also have a larger impact on the lives and economies of more people. Permanent strengthening of high-rise buildings damaged during significant earthquakes, if possible, at all, will be more efficient if the structure can be kept in a stable situation during the aftershocks, allowing the decision makers to take a better-studied action. Here it is suggested to apply a quick strengthening approach without epoxy injection and reinforcement repair for the first time by applying GFRP to damaged coupling beams as a remedial work.

The primary finding of the experiments is that the damaged specimens which are later repaired and strengthened presented a lower strength value but the same or even higher ductility and deformation capacity. This is the case when even longitudinal or diagonal bars had fractured in the initial experiments, meaning that the method is able to provide the same and even higher deformation capacity, even if the coupling beams underwent significant damage.

It should be noted that the presented study is a quick and non-permanent strengthening method; thus, fire resistance measures are not considered herein. Fabric-reinforced cementitious matrix (FRCM) material, or fire-resistant coating, can be used if fire is also a concern.

## Figures and Tables

**Table 1:** Typical dry fiber properties.

Property	Typical Test Value
Tensile Strength	3.24 GPa (470,000 psi)
Tensile Modulus	72.4 GPa (10.5 × 10^6^ psi)
Ultimate Elongation	4.5%
Density	2.55 g/cm^3^ (0.092 lbs./in.^3^)
Weight per sq. yd.	505 g/m^2^ 14.9 oz.

**Table 2:** Epoxy material properties.

Property	ASTM Method	Typical Test Value
Post Cure (24 h)	ASTM D-4065	82 °C (180 °F)
Tensile Strength	ASTM D-638 Type 1	72.4 MPa (10,500 psi)
Tensile Modulus	ASTM D-638 Type 1	3.18 GPa (461,000 psi)
Elongation Percent	ASTM D-638 Type 1	5.0%
Flexural Strength	ASTM D-790	123.4 MPa (17,900 psi)
Flexural Modulus	ASTM D-790	3.12 GPa (452,000 psi)

**Table 3:** Properties of cement-based repair mortar.

**(a)**		
**Color**	**Application Thickness**	**Compression Strength**
Grey	5–50 mm	55 MPa (28 Days)
**(b)**		
**Tensile Strength in Bending**	**Usage Period**	**Floor Temperature to be Applied**
9 MPa (28 Days)	30 min	(+5 °C)–(+35 °C)
**(c)**		
**Dry Powder Density**	**Wet Mortar Density**	**Consumption for 1 cm Thickness**
1.55 kg/L	2.10 kg/L	21 kg/m^2^

**Table 4:** Ductility index values of specimens before damage (without wrapping) and after damage (with GFRP wrapping).

Specimen	Yield Chord Rotation (%)	Ultimate Chord Rotation (%)	Ductility Index Ratio
PCB	1.34	3	2.23
PCB-R	1.25	5	4
UCB	1.35	6	4.44
UCB-R	1.34	6	4.47
RCR	1.43	5	3.50
RCR-R	1.71	6	3.51

**Table 5:** Residual displacement values of specimens before and after GFRP wrapping.

Specimen	Residual Displacement (mm) *
PCB	−9.7
PCB-R	−29.3
UCB	−30.8
UCB-R	53
RCR	26.3
RCR-R	−36.8

* See Figure 4 for positive and negative direction acceptance. (“pull” direction is positive).

## Data Availability

The data used to support the findings of this study are included within the article.
